# Chromosome length genome assembly of the redbanded stink bug, *Piezodorus guildinii* (Westwood)

**DOI:** 10.1186/s13104-022-05924-5

**Published:** 2022-03-22

**Authors:** Surya Saha, K. Clint Allen, Lukas A. Mueller, Gadi V. P. Reddy, Omaththage P. Perera

**Affiliations:** 1 Boyce Thompson Institute, 533 Tower Road, Ithaca, NY 14853 USA; 2 Southern Insect Management Research Unit, USDA ARS, Stoneville, MS 38776 USA

**Keywords:** Redbanded stink bug, *Piezodorus guildinii*, Genome assembly, Pentatomidae, Soybean pest

## Abstract

**Objective:**

The redbanded stink bug (RBSB), *Piezodorus guildinii* (Hemiptera: Pentatomidae), is native to the Caribbean Basin and is currently considered an invasive pest in Florida, Louisiana, Mississippi, and Texas in the southern United States. Although RBSB is an economically important invasive pest in the USA, relatively few studies have been conducted to understand molecular mechanisms, population genetic structure, and the genetic basis of resistance to insecticides. The objective of this work was to obtain a high-quality genome assembly to develop genomic resources to conduct population genetic, genomic, and physiological studies of the RBSB.

**Results:**

The genome of RBSB was sequenced with Pacific Biosciences technology followed by two rounds of scaffolding using Chicago libraries and HiC proximity ligation to obtain a high-quality assembly. The genome assembly contained 800 scaffolds larger than 1 kbp and the N50 was 170.84 Mbp. The largest scaffold was 222.22 Mbp and 90% of the genome was included in the 7 scaffolds larger than 118 Mbp. The number of megabase scaffolds also matched the number of chromosomes in this insect. The genome sequence will facilitate the development of resources to conduct studies on genetics, transcriptomics, and physiology of RBSB.

**Supplementary Information:**

The online version contains supplementary material available at 10.1186/s13104-022-05924-5.

## Introduction

**Fig. 1:**
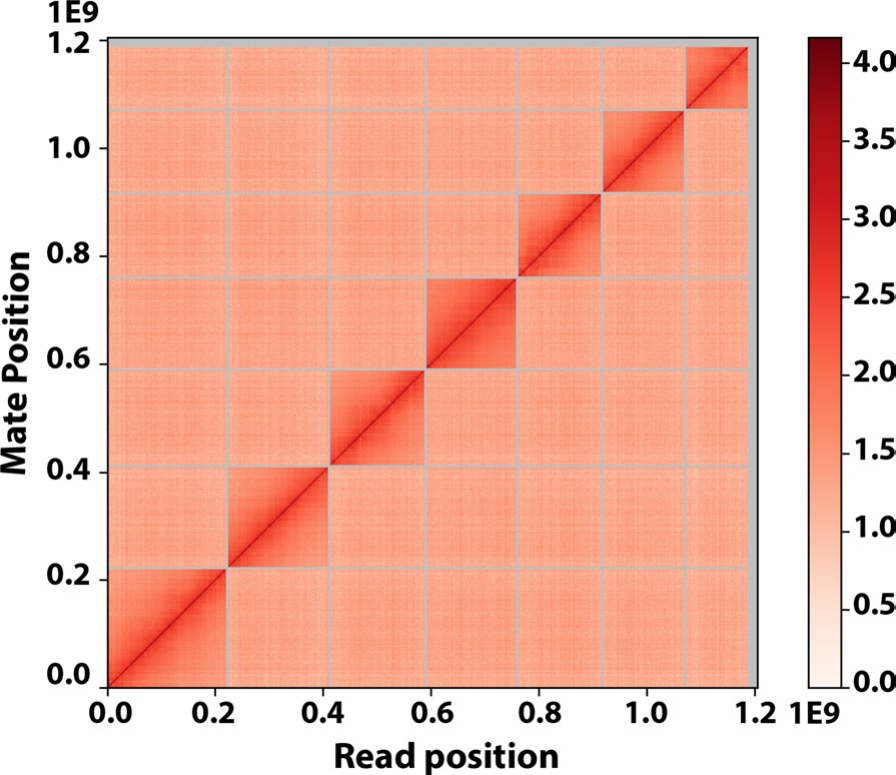
HiC linkage density histogram of *Piezodorus guildinii* genome assembly. The x and y axes in this histogram show the mapping positions of the first and second read in a read pair, respectively, grouped into bins. The intensity of color of each square represents the number of read pairs within that bin. Scaffolds less than 1 Mb were excluded from this histogram

The redbanded stink bug (RBSB), *Piezodorus guildinii* (Westwood) (Hemiptera: Pentatomidae), is native to the Caribbean Basin and is currently considered an invasive pest of soybeans and several other commercially grown crops in Florida, Louisiana, Mississippi, and Texas in the southern United States [1–3]. Uncontrolled outbreaks of RBSB can cause significant economic damage to soybeans from early seed development stages to mature seeds [4]. Although RBSB is an economically important invasive pest in the USA, relatively few studies have been conducted to understand genetic, population genetic structure, and genetic basis of resistance to insecticides. Biology, ecology, host plants, and pest status of this insect has been previously studied [2, 5–12]. Resistance to insecticides in RBSB have been documented [13], but the genetic basis of the insecticide resistance in this insect is not well understood. So far, only one population genetic study has been carried out on this species using 1,337 SNP markers that identified the presence of genetic structure separating populations in USA and Brazil [14]. In order to develop genetic resources to conduct functional genomic, population genetic, and physiological studies, we sequenced the genome of RBSB (Additional file 1: Fig. S1) with Pacific Biosciences long read technology using Chicago libraries and then we assembled the draft input assembly that was used for scaffolding Illumina short reads from HiC proximity ligation libraries to obtain a high-quality assembly (Fig. 1, Additional file 1: Figs. S2 and S3).

## Main text

### Methods

Genomic DNA libraries for PacBio sequencing and initial assemblies were prepared by Dovetail Genomics (Scottes Valley, CA, USA). A Qiagen tissue kit and a tip-20 mini column (Qiagen, Germantown, MD) was used to isolate high molecular weight DNA from a field collected female RBSB. DNA was quantified using Qubit 2.0 fluorometer (Life Technologies, Carlsbad, CA) and a PacBio SMRTbell library with approximately 20 kbp was constructed using SMRTbell Express Template Prep Kit 2.0 (PacBio, Menlo Park, CA) following the manufacturer’s protocol. DNA sequencing was performed on a PacBio Sequel II sequencer using Sequel II 8 M SMRT cells generating 108 Gb of continuous long reads (CLR). Initial assembly was performed using the Wtdbg2 v.2.5. assembler [15] with the following parameters: --genome_size 1.0 g --read_type sq --min_read_len 20,000 --min_aln_len 8192. Blob Tools v1.1.1 [16] with default parameters ([-t HITS…] [-x TAXRULE…] [-m 0.0] [-d 0.0] [–tax_collision_random]) was used to identify potential contamination based on BLAST (v2.9) results of the assembly against the NT database. The assembly was filtered to remove potential haplotypic duplications using Purge Dups v1.1.2 [17] (parameters: -2 -T cutoffs) to obtain a purged draft assembly used for scaffolding. Scaffolding was performed using HiRise v2.1.5 pipeline [18] with default settings. Proximity ligation libraries were prepared using Dovetail Omni-C library protocol by digesting formaldehyde fixed chromatin with a DNAse I, repairing chromatin ends, and biotinylated bridge adapter ligation followed by proximity ligation of adapter containing ends. Then crosslinks were reversed, DNA was purified and was treated to remove biotin that was not internal to ligated fragments. Biotin-labelled DNA fragments were isolated using streptavidin beads and enriched by PCR. The library was sequenced on an Illumina HiSeqX platform to produce approximately 30 × sequence coverage. The draft de novo assembly from PacBio reads and Dovetail Omni-C proximity ligated library reads were used as input data for HiRise v2.1.5 pipeline. Dovetail Omni-C library sequences were aligned to draft input assembly using BWA-mem v0.7.17-r1188 [19, 20] using parameters 5SP -T0. The separations of Dovetail OmniC read pairs mapped within draft scaffolds were analyzed by HiRise to produce a likelihood model for genomic distance between read pairs, and the model was used to identify and break putative mis-joins, to score prospective joins, and make joins above a threshold.

The genome completeness evaluation was performed with BUSCO version 5.2.2 [21] using the Arthropoda (arthropoda_odb10.2019-11-20 1013) and Hemiptera (hemiptera_odb10.2019-11-20 2510) databases with 1,013 and 2,510 BUSCOs, respectively. The repeat annotation was done with RepeatModeler using the Dfam TE tools docker container version 1.4 (https://github.com/Dfam-consortium/TETools). The repeat classification was performed with RepeatClassifier Version 2.0.2 and RepeatMasker version 4.1.2-p1 [22] to identify the types of repeats in the RBSB genome. RepeatMasker was run in sensitive mode with rmblastn version 2.11.0 + . The database of repeats used for classification was Dfam 3.4 [23].

To identify reads derived from mitochondrial DNA (mtDNA), PacBio sequence reads from the shotgun library were mapped to the *Nezara viridula* (L.) mtDNA genome (Accession: EF208087.1) with relaxed parameters (length fraction 0.3 and similarity fraction 0.75). Mapped sequence reads were extracted, and de novo assembled at 85% similarity and 75% length fraction. A consensus of a 20,764 nt RBSB mtDNA contig with 84% identity to the stink bug *Eurydema ventralis* (Kolenati) mtDNA (Accession:MG584837.1) was selected for use as the reference to map the PacBio reads with a higher stringency at 85% similarity fraction and 75% length fraction. A total of 2,099 out of 9,113,332 reads were mapped to the RBSB mtDNA reference with > 150-fold sequence coverage across the coding regions and > 75-fold sequence coverage in the control region. Consensus of this contig was used to obtain 18,889 bp mitochondrial genome of RBSB.

### Results and discussion

Published and unpublished genome assemblies of other pentatomids available in databases indicate that the RBSB genome assembly presented here is comparable to chromosome length assembly of *Aelia acuminata* (N50 = 172.2 Mbp, largest scaffold = 235.2 Mbp; [24]) and superior to the assemblies of *Euschistus heros* (N50 = 2.46 Mbp; PRJNA489772), and *Halyomorpha halys* (N50 = 802 Kbp; [25]). The assembled size of the RBSB genome was 1.205 Gbp with an N50 of 170.835 Mbp, N90 of 118.462 Mbp, L50 of 4, and L90 of 7. The final genome assembly contained 800 scaffolds larger than 1 Kbp and the smallest and the largest scaffold were 118.462 and 222.218 Mbp, respectively (Table 1). Karyotyping of RBSB identified six pairs of autosomes and a pair of sex chromosomes designated X and Y (2n = 14) [26]. The seven largest scaffolds in the RBSB genome assembly matched the haploid chromosome number in RBSB and other pentatomid bugs.

**Table 1:** Assembly summary and contiguity metrics for the shotgun input assembly of PacBio reads and the final scaffolding with proximity ligation (HiC) of the RBSB genome

	Input assembly	Dovetail HiRise assembly
Total length (bp)	1,205,371,278	1,205,416,778
N50	7,401,307	170,835,737
L50	46	4
Largest scaffold	29,620,976	222,218,125
Number of scaffolds	1262	807
Number of scaffolds > 1kbp	1255	800
Number of gaps	5	460
Number of N's per 100 kbp	0.01	3.78

The genome assembly of RBSB was also found to be highly complete for single-copy markers conserved within the Arthropoda and Hemiptera clades with 96.5% and 96.2% completeness values (Table 2). The low duplication and fragmentation coupled with the completeness underlines the integrity of this genome assembly.

**Table 2:** BUSCO completeness statistics on the Arthropoda and Hemiptera marker set for the RBSB genome assembly

BUSCO database	Complete BUSCOs	Complete and single-copy BUSCOs	Complete and duplicated BUSCOs	Fragmented BUSCOs	Missing BUSCOs	Total BUSCO groups searched
Arthropoda	977 (96.5%)	962 (95%)	15 (1.5%)	10 (1%)	26 (2.5%)	1013
Hemiptera	2414 (96.2%)	2375 (94.6%)	39 (1.6%)	21 (0.8%)	75 (3%)	2510

These numbers were generated with BUSCO version 5.2.2

We performed two rounds of repeat annotations for the RBSB genome. It was analyzed for known repeat families in Insecta present in the DFAM 2.4 [27] (Additional file 1: Table S1). LINE (11.64%) were the predominant retroelements found followed by SINEs (1.21%). The Tc1-IS630-Pogo transposon was the predominant DNA transposon repeat family (4.51%). A de novo repeat annotation using RepeatModeler [28] identified 2338 RepeatScout/RECON families and 181 LTR repeat families. All annotations are available to the research community at the AgriVectors portal [29].

The complete sequence of the mitochondrial genome, which is often used in population genetics and molecular identification of insects is not currently available for this species. Availability of the complete mitochondrial genome assembled in this study (BioSample SAMN23701154) will provide additional DNA markers to conduct population studies that require relatively highly variable mitochondrial genes such as NADH dehydrogenases (*ND*) and cytochrome B.

Additionally, having an official gene set predicted by the NCBI eukaryotic annotation pipeline (https://www.ncbi.nlm.nih.gov/genome/annotation_euk/process/) based on this high-quality genome will aid in conducting expression profiling experiments intended to elucidate physiological responses to various host plant species and insecticides. Large scale insect genomics projects like the i5k [30] and more recently Ag100Pest [31] have also highlighted the long-term benefits of building open access databases and genomics resources for the community.

A limited number of molecular resources are currently available for RBSB in public databases, which include 107 microsatellite sequences, six partial cytochrome oxidase I (*COI*) subunit sequences [32], a 3.18 Mbp partial assembly with 1932 genomic contigs (N50 = 1494 bp; PRJNA263369), and 17 Gbp of genotype by sequencing (GBS) data in the NCBI Sequence Read Archive [14]. Transcriptome, genome, or proteome data are not available for this economically important pest species. Besides the high-quality genome assembly, we have generated triplicate RNASeq libraries for all life stages from eggs, first instar to fifth instar nymphs, and adult males and females that will be sequenced to obtain a minimum coverage of 25 million (2 × 150 paired end) reads for each library. Oxford Nanopore long reads will also be obtained by pooling mRNA from all life stages of RBSB. Long Oxford Nanopore reads will facilitate annotation of full-length gene models in the genome as well as qualitative identification of isoforms from each life stage. The NCBI structural annotation will be followed by functional annotation to identify gene ontology terms and pathways[33] which will be made available on the AgriVectors portal [29]. In addition, we plan to sequence the RBSB genomic DNA with Oxford Nanopore reads to identify epigenetic modifications such as methylation in the genome [34, 35].

Having a well characterized high quality genome assembly will provide a more robust foundation for developing genetic markers for population genetic studies, linkage mapping, and identifying genomic regions associated with regulation of gene expression, host selection, and insecticide resistance by comparative genomic studies using published genomes and transcriptomes of other insects and pentatomid stink bugs [24, 25, 36–38].

### Limitations

Genomic DNA library preparation and assembly were performed with proprietary methods developed by a service provider with PacBio and Illumina sequencing versions currently available. Library construction and sequencing methods, assembly software, and other data used for comparative analysis may be updated in the future. PacBio continuous long reads (CLR) may contain insertion and deletion errors, some of which may have escaped correction during the assembly process.

## Supplementary Information

**Additional file 1: Figure S1.** Female (top left) and male (top right) of *Piezodorus* *guildinii*. **Figure S2.** Comparison of the cumulative lengths of the purged input assembly and the final HiRise scaffolds. **Figure S3.** Cumulative insert size distribution of HiC paired-end reads mapped within a chromosome. **Table S1**. The number of repeat units, the total length and the percentage of different repeat families identified in *Piezodorus guildinii* genome. 

## Data Availability

All raw sequencing data and assemblies have been submitted to NCBI BioProject PRJNA686660.
