# Supplementary material for: Chromosome length genome assembly of the redbanded stink bug, Piezodorus guildinii (Westwood)
Source: BMC Res Notes. 2022 Mar 22;15:115. doi: 10.1186/s13104-022-05924-5 (PMC8939125; doi:10.1186/s13104-022-05924-5)
Supplement: Supplementary file 1 — Additional file 1: Figure S1. Female (top left) and male (top right) of Piezodorus guildinii. Figure S2. Comparison of the cumulative lengths of the purged input assembly and the final HiRise scaffolds. Figure S3. Cumulative insert size distribution of HiC paired-end reads mapped within a chromosome. Table S1. The number of repeat units, the total length and the percentage of different repeat families identified in Piezodorus guildinii genome. [file 13104_2022_5924_MOESM1_ESM.docx]

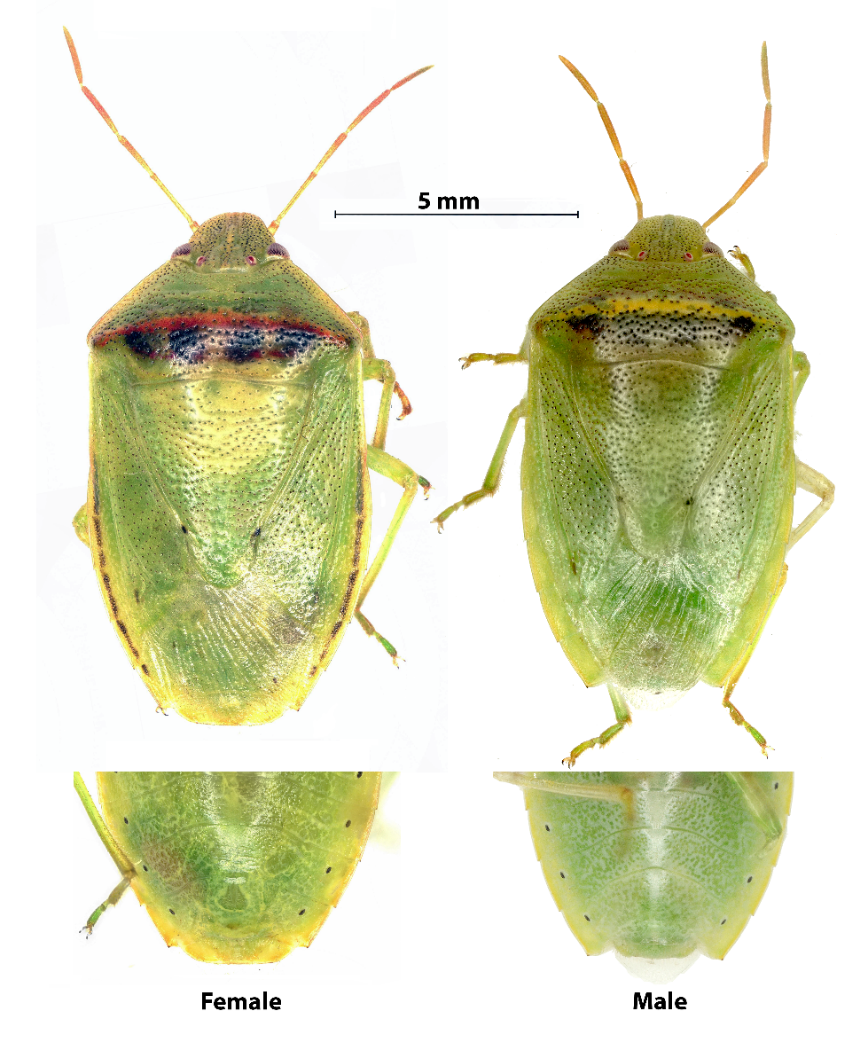


 Figure S1. Female (top left) and male (top right) of *Piezodorus guildinii*. Color of the transverse band on the pronotum and the lateral bands on the edges of wing covers may vary from dark red to light yellow in both sexes. Tips of the abdomen of female and male are shown on the bottom left and bottom right, respectively.


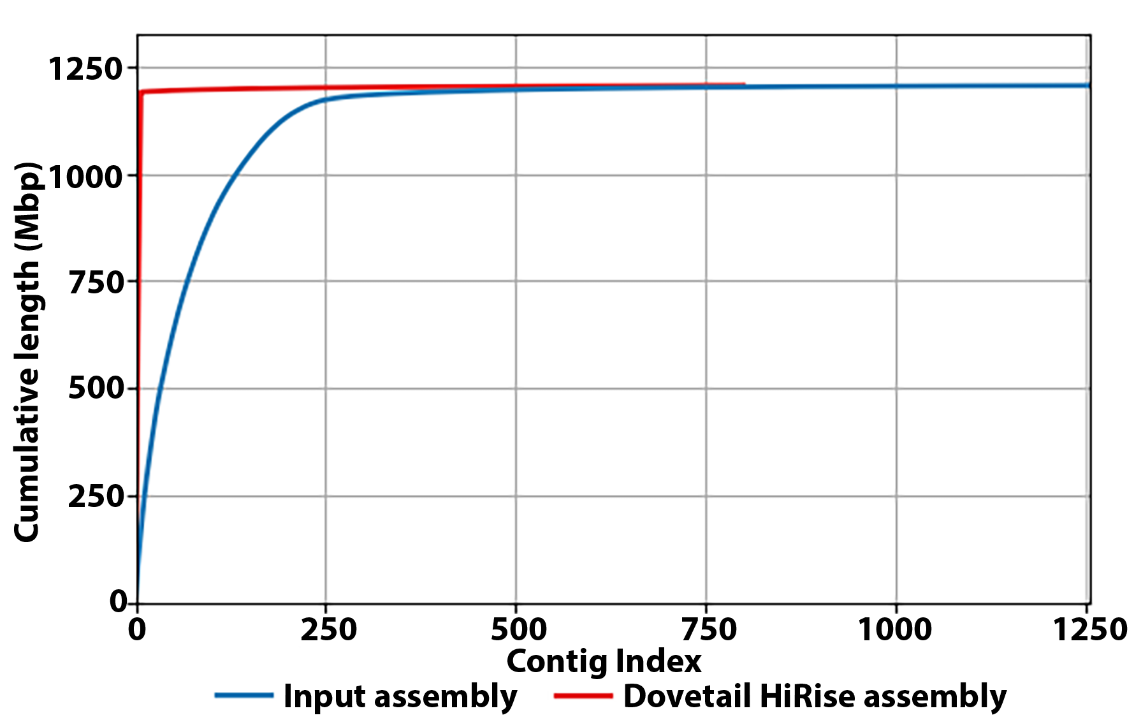


Figure S2. Comparison of the cumulative lengths of the purged input assembly and the final HiRise scaffolds. The scaffold number is given on the X-axis and the cumulative length of each assembly is indicated on the Y-axis. Scaffolds less than 1 kb were excluded.


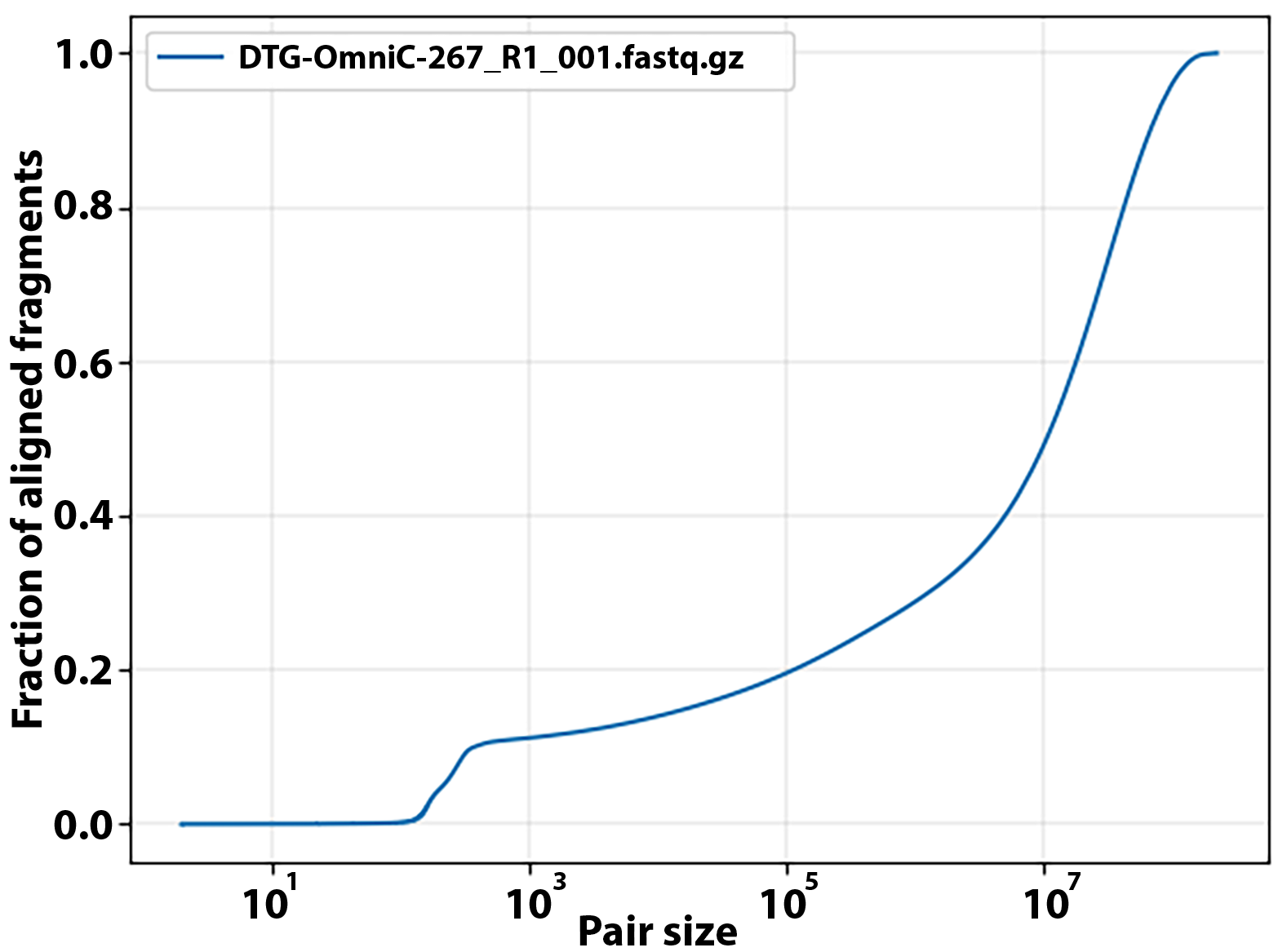


Figure S3. Cumulative insert size distribution of HiC paired-end reads mapped within a chromosome.

Table S1: The number of repeat units, the total length and the percentage of different repeat families identified in *Piezodorus guildinii* genome. The query species was assumed to be Insecta. RepeatMasker version 4.1.2-p1 was run in sensitive mode with rmblastn version 2.11.0+ and FamDB: CONS-Dfam_3.4.

|  | Number of elements | Total length | Percentage |
| --- | --- | --- | --- |
| Retroelements | 447888 | 167467876bp | 13.89% |
| SINEs: | 73583 | 14645199bp | 1.21% |
| Penelope | 11344 | 3990227bp | 0.33% |
| LINEs: | 350888 | 140295032bp | 11.64% |
| L2/CR1/Rex | 84909 | 29193274bp | 2.42% |
| R1/LOA/Jockey | 27485 | 10677575bp | 0.89% |
| R2/R4/NeSL | 6843 | 2799554bp | 0.23% |
| RTE/Bov-B | 105327 | 45712074bp | 3.79% |
| L1/CIN4 | 1711 | 157553bp | 0.01% |
| LTR | elements: | 23417 | 12527645bp |
|  |  |  |  |
| BEL/Pao | 1509 | 640075bp | 0.05% |
| Ty1/Copia | 492 | 68760bp | 0.01% |
| Gypsy/DIRS1 | 21290 | 11768227bp | 0.98% |
|  | | | |
| DNA transposons | 252860 | 57500976bp | 4.77% |
| hobo-Activator | 3224 | 584070bp | 0.05% |
| Tc1-IS630-Pogo | 234671 | 54408626bp | 4.51% |
| PiggyBac | 229 | 25517bp | 0.00% |
| Tourist/Harbinger | 706 | 322058bp | 0.03% |
| Rolling-circles | 238017 | 60691804bp | 5.03% |
| Unclassified: | 696974 | 128340103bp | 10.65% |
| Total interspersed repeats: |  | 353308955bp | 29.31% |
| Small RNA: | 60129 | 11935927bp | 0.99% |
| Satellites: | 994 | 121815bp | 0.01% |
| Simple repeats: | 664320 | 29556395bp | 2.45% |
| Low complexity: | 112145 | 5583458bp | 0.46% |
